# Effect of Ultraviolet Aging on Properties of Epoxy Resin and Its Pultruded Fiber-Reinforced Composite

**DOI:** 10.3390/polym17030294

**Published:** 2025-01-23

**Authors:** Shengzong Ci, Baoming Wang, Chengrui Di, Mingyu Wang, Bo Zhu, Kun Qiao

**Affiliations:** 1 Weihai Guangwei Composites Co., Ltd., Weihai 264202, China; wangbm2008@126.com; 2 Weihai Research Institute of Industrial Technology, Shandong University, Weihai 264209, China; cishengzong57379@126.com (S.C.); dicr0918@163.com (C.D.); wangmy0730@163.com (M.W.); 3 Shandong Key Laboratory of Carbon Fiber and Composite Materials Manufacture and Application, Shandong University, Weihai 264211, China; 4 Fiber and Composite Engineering Center, Weihai Research Institute of Industrial Technology, Shandong University, Weihai 264211, China

**Keywords:** polymer matrix composites (PMCs), environmental degradation, ultraviolet irradiation, mechanical properties

## Abstract

Polymer matrix composites (PMCs) often undergo aging as a result of ultraviolet (UV) radiation, which significantly impacts their performance and durability. This paper investigated the alterations in the microstructure and macroscopic properties of epoxy resin and its composite used in overhead wires during UV aging. Furthermore, the mechanism of UV aging for both resin and composite was revealed. The results showed that UV aging predominantly affected the properties of the surface layer resin. UV aging can induce molecular chain scission, which leads to resin weight change, color deepening, microcrack formation, a decline in mechanical properties, and other performance degradation behaviors under the combined action of many factors. With the increase in aging time, the weight change rate and hardness of the resin increased first and then decreased, while the mechanical properties of the composite decreased rapidly first and gradually tended to be constant. The bending strength and impact strength of the composite decreased by 6.0% and 12.8%, respectively, compared with the initial values. The purpose of this study is to comprehensively understand the UV aging behaviors of epoxy resins and their composites employed in overhead wires, and it also provides essential data for advancing the utilization and durability of epoxy resins and composites across aerospace, marine, and other outdoor applications.

## 1. Introduction

Carbon fiber reinforced polymer (CFRP) has been widely used in the fields of military and civilian due to its advantages of high strength and high modulus, lightweight, heat resistance, impact resistance, and wear resistance [1,2,3]. Epoxy, as one of the prominent thermoset polymer matrices characterized by its irreversible cross-linking structure formed during curing, is renowned for its excellent adhesion properties, enabling it to bond with carbon fibers effectively, thereby enhancing the overall mechanical performance of the composite. Pultrusion is a processing technology that shapes composites into the desired cross-sectional profiles, such as rods, tubes, or other custom forms through the die. This technique not only ensures the uniform distribution of fibers, which maximizes their strength and stiffness contribution to the final CFRP products but also enables their high-volume production.

With the expanding application domains, the service environment of composites has become increasingly intricate and severe, encompassing extreme temperatures, precipitation including rain and snowfall, wind sand, microorganisms, and seawater corrosion [4,5,6,7,8]. To ensure the safety and reliability of composite products, researchers have conducted a lot of research on their environmental durability.

Ultraviolet (UV) irradiation is potentially amongst the most damaging weathering conditions that affect material properties deleteriously. It is also a crucial research direction for the environmental degradation of materials [9,10,11,12,13]. Polymeric materials undergo photo-oxidation reactions, thermal oxidation, and photolysis reactions when exposed to UV light [14]. Many studies have demonstrated that they exhibit destructive characteristics towards the polymers, initiating a chain scission process. Then, chain scission at the polymer surface causes microcrack formation, diminishes color, and leads to covalent bond breakage, hence decreasing the mechanical properties of materials [15,16]. Ahmad et al. [17] confirmed that microcracks and discoloration appeared on the surface of the epoxy coatings after 1000 h of UV exposure. Wang et al. [18] investigated the UV chemical aging and hydrolysis processes of polyolefin (PE) and polyester (PLA) microplastics in aqueous environments and analyzed and clarified their degradation trends and the characteristics of the degradation products. Compared with polymers, composites have a more complex composition, typically containing multiple components with different properties, such as fiber reinforcement phases, resin matrices, and various additives. When composites are exposed to UV radiation, different components vary in their absorption, scattering, and response mechanisms to UV light. This makes the UV aging mechanism of composite far more intricate than that of polymers. Cao et al. [19] evaluated the UV aging behaviors of resin and GFRP laminates with different curing agents, including polyamide, alicyclic, and phenolic amine curing agents. Deng et al.’s study results [20] indicate that the CFRP shows noticeable synergistic aging effects when simultaneously exposed to UV radiation and a salt-fog environment. The decrease in the cross-linking density of CFRP is the primary cause of the deformation resistance reduction and properties degradation. Zhao et al. [9] established the multiscale finite element model of the R/C composites (ramie fiber and carbon-fiber-reinforced polyethylene terephthalate glycol) to study the UV aging mechanism and predict their mechanical degradation. Therefore, investigating the effects of UV irradiation holds great significance. This is because UV irradiation can notably degrade the performance of composites, undermining their mechanical properties, durability, and overall integrity, thus making in-depth research on this topic crucial for enhancing the long-term reliability and service life of composites and structures.

**Figure 1:**
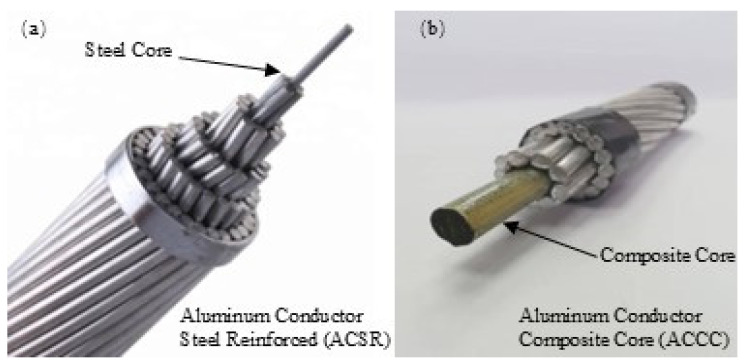
Overhead transmission lines with different reinforcement cores. (**a**) Aluminum Conductor Steel Reinforced (ACSR); (**b**) Aluminum Conductor Composite Core (ACCC).

Aluminum Conductor Composite Core (ACCC) is a new type of wire for overhead transmission lines, which uses the composite core to replace the steel core of Aluminum Conductor Steel Reinforced (ACSR) [21], as shown in Figure 1. The prolonged exposure of wires to the outdoor environment renders polymer-based reinforced materials highly susceptible to light radiation, thereby posing a significant threat to the secure operation of power grid lines. The application time of ACCC is still relatively short, thus the research on its long-term operational properties under actual complex conditions is primarily in its nascent stage or even lacking, resulting in a dearth of guiding data for accurately assessing performance and conducting operational maintenance of the product during practical usage. Hence, it is particularly important to research the weather resistance of resin and composite materials for the ACCC, which is of great significance to ensure the safety and promotion of composite products.

This study focuses on epoxy resin and composite dedicated to the composite core of ACCC. Artificial accelerated ultraviolet aging was employed to simulate the exposure of ACCC to UV radiation. The evolution laws of the macroscopic properties of the epoxy resin and composite under different UV aging conditions were systematically studied, including changes in mechanical and physical properties. The internal relationships among UV aging, internal structure, and macroscopic properties of the materials were revealed. It provides a new theoretical basis and technical support for enhancing the long-term safety and reliability of carbon fiber composite cores in the field of power transmission. It also provides reference data for other outdoor applications of composites.

## 2. Materials and Methods

### 2.1. Materials

An epoxy resin with multi-functional groups and an anhydride curing agent was sourced from DOW chemical company, Midland, MI, USA. Six-membered heterocyclic benzo compounds, the toughening modifier, defoamer, release agent, and other reagents were developed by the Carbon Fiber Engineering Technology Research Centre of Shandong University, Jinan, China. The toughening modifier was a compound possessing two active end groups, wherein the chain segments were interconnected via ester bonds. Upon curing, it dispersed within the cured epoxy resin in the form of spherical micro-particles, forming a structure similar to “sea island”, thereby achieving the purpose of toughening. Carbon fiber (T700-12K-50C) was sourced from Toray Industries, Inc., Tokyo, Japan. High-strength bidirectional glass fiber tape was purchased from Shandong Lufa Carbon Fiber Composites Co., Jinan, China. Other reagents, including sodium chloride, anhydrous ethanol, and acetone, were commercially obtained in China.

### 2.2. Sample Preparation

#### 2.2.1. Preparation of Epoxy Resin Castings

**Figure 2:**
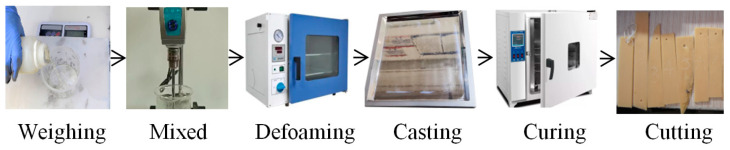
Flow chart of resin sample preparation.

Epoxy resin, curing agent, modifier, and defoamer were mixed in a fixed weight proportion of 100:100:30:6, respectively, to form a gel with a viscosity of approximately 300~500 mPa·s (25 °C). After vacuum defoaming, the gel was cured and shaped into a 3 mm thick casting sheet under the curing procedure of 80 °C/1 h + 100 °C/2 h + 160 °C/2 h. The epoxy sheet was then cut into several sample strips. The process is schematically shown in Figure 2. The epoxy sample strips used in the aging study were cut from the same sheet to ensure uniformity of performance and color.

#### 2.2.2. Preparation of Carbon Fiber/Glass Fiber Hybrid Reinforced Epoxy Pultruded Composites

**Figure 3:**
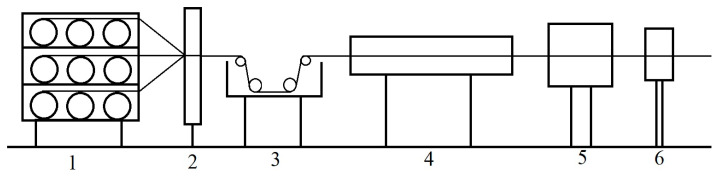
Schematic of pultrusion molding (1. Creel; 2. fiber guide; 3. resin impregnator; 4. mold; 5. tractor; 6. cutter.).

Composite rods with a diameter of 7.5 mm were prepared using a self-developed pultruding process. The matrix used to prepare the composites was the same as that used in the preparation of epoxy castings. The reinforcement included internal unidirectional T700 carbon fiber and external high-strength bidirectional glass fiber cloth, where their volume content was 58% and 2%, respectively. The glass fiber tape and carbon fibers were impregnated in an epoxy resin gel solution at a pulling speed of 500~700 mm/min, followed by curing in a mold at temperatures ranging from 150 to 200 °C. The process is schematically shown in Figure 3. The composite rod samples were collectively referred to as CFRP for the sake of convenience.

### 2.3. Experimental Methods

The UV aging was carried out with the Chinese standard test GB/T 14522-2008 [22] within a dedicated UV aging chamber to simulate the effects of high irradiation environments. A total of 12 h was considered one aging cycle. The typical test condition 6 provided in Appendix C of the standard was selected as the test condition for UV aging. The specific parameters for conducting UV aging are presented in Table 1.

### 2.4. Characterization Methods

The weight change of the epoxy resin was measured by using an electronic balance (AG204, Mettler Toledo, Columbus, OH, USA) with a readability of 0.1 mg. The resin density was determined using the buoyancy method. The hardness change of epoxy resin was determined by a Richter hardness tester (AR936, Sigma Technology, Göteborg, Sweden). Chemical structure changes in the epoxy resin and CFRP were examined via Fourier transform infrared spectroscopy (VERTEX-70, Bruker, Karlsruhe, Germany) at a resolution of 4 cm^−1^ and 32 scans from 4000 to 500 cm^−1^. The FTIR samples were prepared by the potassium bromide tablet method. The powder was polished from the surface located at almost the mid-plane of the samples and then mixed with KBr at a mass ratio of 1:100 to form small pieces of pellets. The thermal decomposition of the epoxy resin was analyzed using a thermogravimetric analyzer (SDT-Q600, TA, New Castle, DE, USA) under a nitrogen atmosphere, with the temperature ranging from 25 to 800 °C at a heating rate of 10 °C/min. The surface morphology characterization of the epoxy resins and their composites was carried out using a scanning electron microscope (SEM, SU-70, Hitachi, Ibaraki, Japan). The three-point bending test and unnotched impact test of CFRP were measured by using the universal testing machine (CMT4204, Shenzhen Sans Testing Machine Co., Ltd., Shenzhen, China) and impact tester (XJJ-50, Chengde Dahua Testing Machine Co., Ltd., Chengde, China), respectively. The length of bending test samples was 120 mm, the support span was 60 mm, and the loading speed was 2 mm/min. The impact sample was directly cut from the composite conductor rod, with a length of 12 cm. Impact strength can be expressed by the following Formula (1):

(1) $$a=\frac{E}{\pi \times r^{2}}\times 10^{3}$$

where a is the impact strength (KJ/m^2^), *E* is the impact energy absorbed by the specimen (J), and *r* is the radius of the sample.

The relevant mechanical properties tests in this paper were usually measured 5 times, and the average value of these measurements was taken as the final result.

## 3. Results and Discussion

### 3.1. Aging Properties of Epoxy Castings

As a crucial component of the composite, resin is the first to be affected by the energy of photons when the composite is exposed to the UV environment for a long time. In this study, a series of experiments and analyses were first carried out to comprehensively investigate the changes in various properties of resin casting plates under UV irradiation, including but not limited to changes in physical properties, mechanical properties, and chemical structure.

#### 3.1.1. Weight and Density

**Figure 4:**
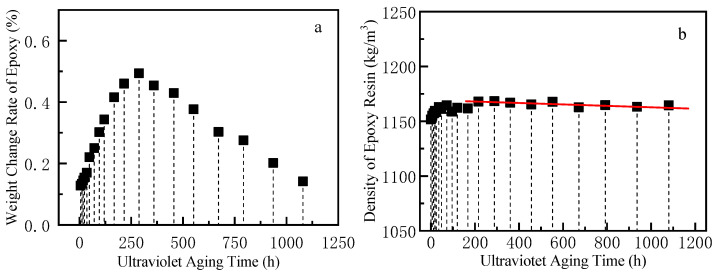
Weight change rate and density change of epoxy after UV aging. (**a**): Weight change rate; (**b**): density.

The weight and density changes in epoxy resin under different UV aging times are shown in Figure 4a and Figure 4b, respectively. Both of them showed a similar law, increasing in the early stage and decreasing in the later stage. The weight change rates of epoxy resin after 12 h, 120 h, 288 h, 792 h, and 1080 h of UV aging were recorded as 0.13%, 0.34%, 0.49%, 0.28%, and 0.14%, respectively. Overall, the change in resin weight was not very significant, but the weight of the aged resin was always higher than that of the initial resin. The resin weight increase during aging was likely due to the absorption of moisture from the environment. Resins can have hygroscopic properties, especially when exposed to ambient conditions over time. The water molecules can interact with the resin matrix, leading to an increase in mass. The initial resin density was about 1151.5 kg/m^3^, which increased to 1164.5 kg/m^3^ after 72 h. Subsequently, there was only a marginal decrease in density.

Under the combined influence of ultraviolet irradiation, spray, and high temperature, resin molecules underwent photooxidation, cross-linking, and degradation processes that coexist and compete with each other. The resin underwent a cross-linking reaction, resulting in an increased degree of cross-linking and making the resin denser. Moreover, surface oxidation at elevated temperatures resulted in an increase in surface density due to the outgassing of volatiles and shrinkage. Photooxidation and hydrolysis reactions may be the main causes of material weight loss [23,24]. Photooxidation of small fragments formed by the cross-linked network of macromolecules and hydrolysis of a limited number of uncross-linked ester bonds together contributed to aging degradation and micro-cracks.

#### 3.1.2. Color

**Figure 5:**
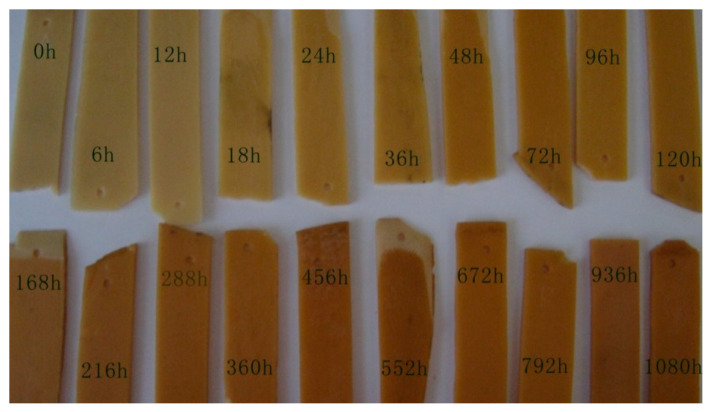
Photo of epoxy after UV aging.

Figure 5 shows the appearance color change of epoxy resin before and after UV aging. As can be seen from the figure, with the progress of ultraviolet aging, the color of the epoxy resin gradually deepened, from light yellow to dark yellow. This phenomenon arises from the degradation of resin molecules upon exposure to ultraviolet radiation, wherein the chain segments of the resin molecule undergo breakage, leading to the generation of active free radicals. These radicals then engage in a photooxidation reaction with atmospheric oxygen, resulting in the formation of new chromophore groups and subsequent alteration in sample color [25]. Some scholars have suggested that the yellowing of resin color is associated with the formation of carbon–oxygen double bonds [26,27].

**Figure 6:**
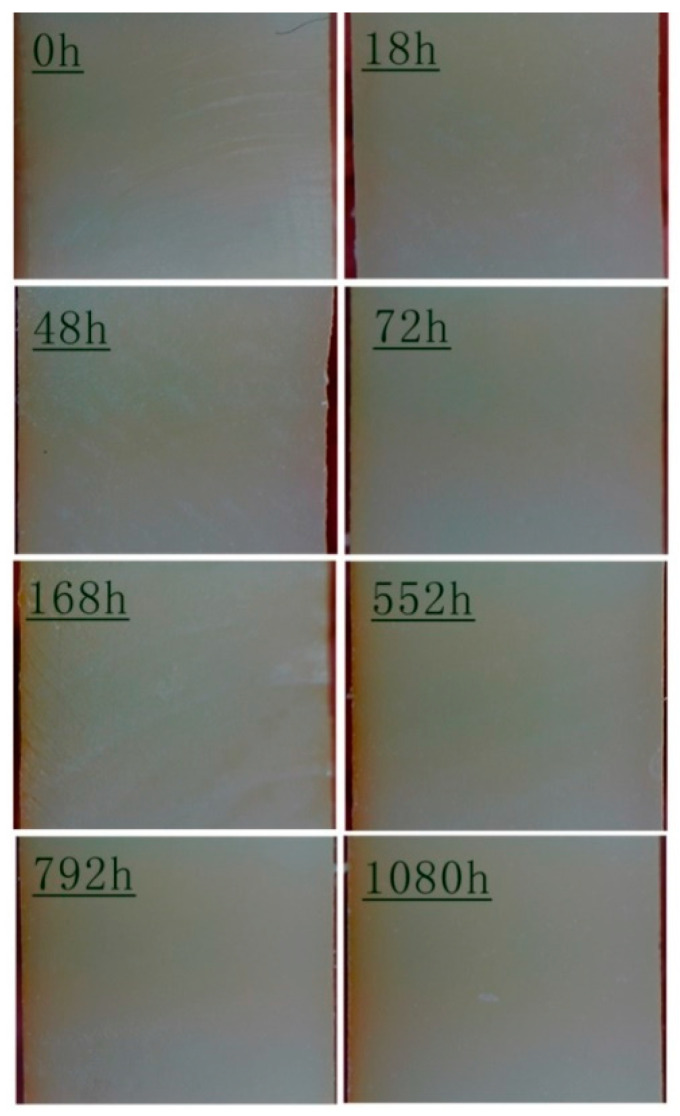
Cross-sectional images of the epoxy after UV aging.

The cross-sectional color change of the epoxy before and after aging is illustrated in Figure 6. As depicted, no significant color change can be observed before 48 h. However, a slight yellowing appeared only on the outermost layer of the resin cross-section after 72 h. As the duration of UV aging increased, the color of the resin’s outermost layer gradually deepened and began to gradually spread inwards. This occurred because UV light propagated from the exterior to the interior via processes such as reflection, absorption, and scattering. Nevertheless, the depth of transmission and the energy ultimately reaching the interior was restricted. Consequently, UV aging had a primary impact on the properties of the surface-layer resin.

#### 3.1.3. Hardness

**Figure 7:**
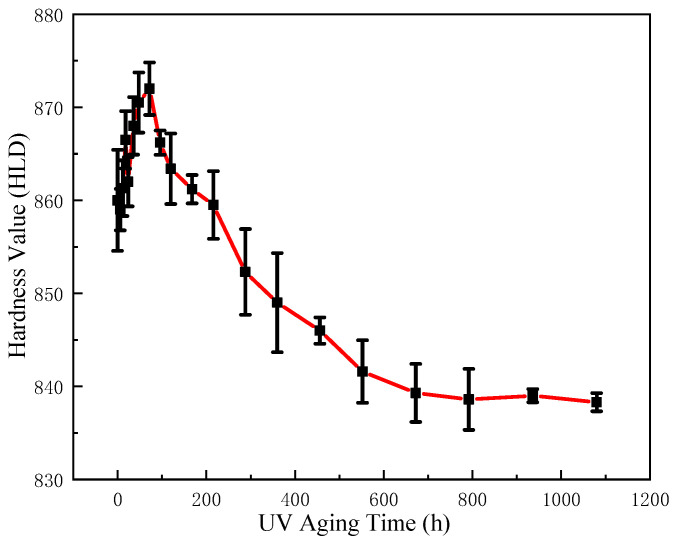
Hardness change of epoxy before and after UV aging.

The change in the Leeb hardness of epoxy resin before and after UV aging is illustrated in Figure 7. As depicted, the Leeb hardness of epoxy resin exhibits a gradual increase during the initial stages of UV aging, reaching a maximum value of 872 HLD at 72 h, representing a 1.4% increment compared to the non-aged epoxy hardness value of 860 HLD. The cross-linking degree of the resin increased at this early stage of UV aging due to the influence of ultraviolet radiation, resulting in a denser structure and, consequently, an increase in hardness. With the progression of UV aging, the resin exhibited cracking and surface defects, accompanied by a decrease in hardness with values recorded as follows: 861 HLD at 168 h, 849 HLD at 360 h, and 838 HLD at 792 h. Thereafter, the hardness of the resin remained at about 838 HLD and did not continue to decline. This stabilization occurred because the UV aging radiation depth and the Leeway hardness test depth were relatively shallow.

#### 3.1.4. FTIR Analysis

**Figure 8:**
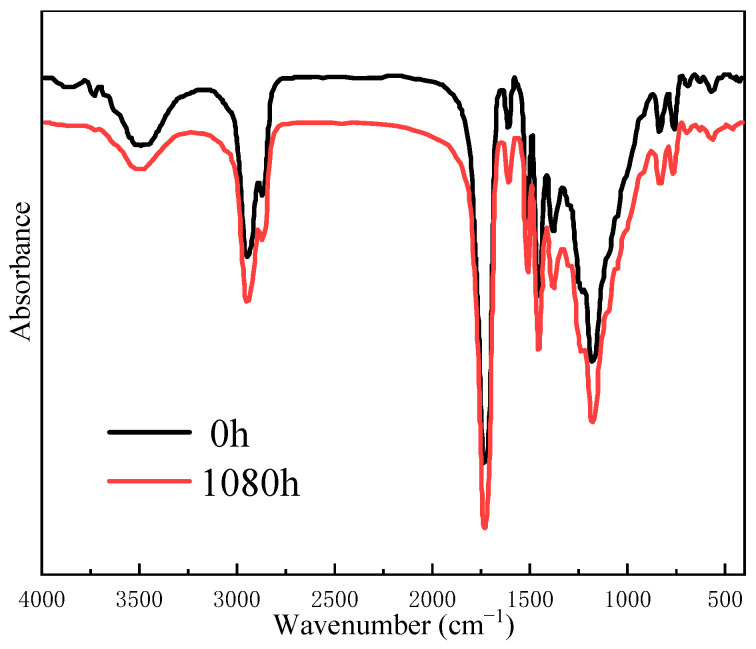
FTIR patterns of epoxy before and after UV aging.

Figure 8 shows the FTIR patterns of epoxy before and after UV aging. It can be seen from the spectrum that before and after UV aging, the position of all characteristic peaks of the epoxy remained unchanged, whereas some peaks’ intensity exhibited an increase or decrease in variation. Notably, the intensity of the characteristic peak, which is attributed to -OH at 3500 cm^−1^, decreased, while the intensity of characteristic peaks, which are attributed to the stretching vibrations of the carbonyl group and ether bond at 1729 cm^−1^ and 1180 cm^−1^, respectively, increased. This indicated that the hydroxyl group in the epoxy resin may be oxidized or etherified under the combination of ultraviolet irradiation, oxygen, and water.

#### 3.1.5. TG and DTG

**Figure 9:**
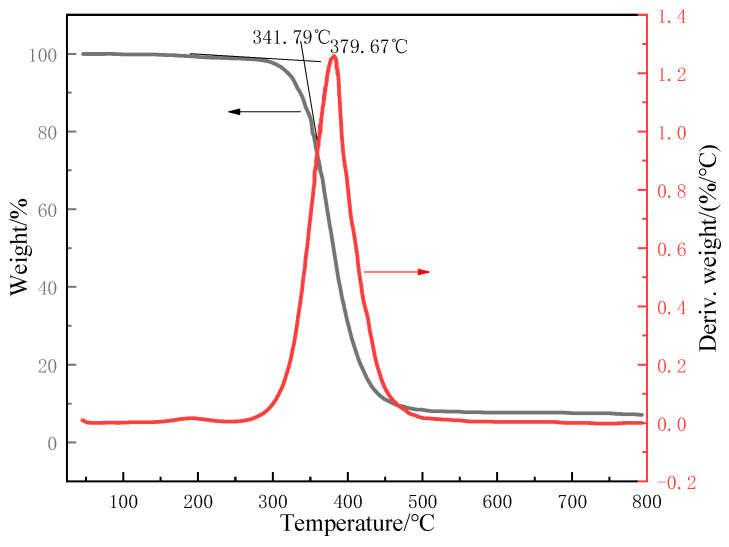
TG and DTG curve of unaged epoxy resin.

The TG and DTG curves of unaged epoxy resin are presented in Figure 9. From the overall trend of the TG curve, it can be observed that the weight of the epoxy resin presented a phased variation with the temperature rising. In the temperature range of 50 °C to 250 °C of the TG curve, the resin’s weight exhibited a slight downward trend. Simultaneously, a weak peak emerged at around 200 °C on the corresponding DTG curve, which could be attributed to multiple factors: (1) If the epoxy resin was not fully cured during the previous curing process, a post-curing reaction may occur at around 200 °C. This process may be accompanied by heat release and mass changes. (2) At elevated temperatures, the chemical bonds within the epoxy resin can react with atmospheric oxygen, leading to the cleavage of molecular chains and the formation of volatile decomposition products, which results in a reduction in the sample’s mass. (3) The epoxy resin system contains some small-molecule substances, such as unreacted monomers or additives. When heated to around 200 °C, the volatility of these small-molecule substances increased, and they began to escape from the sample. (4) At around 200 °C, the molecular segments of the epoxy resin may gain sufficient energy to undergo relaxation and rearrangement. The movement of these molecular segments may lead to changes in the physical structure of the sample, thereby causing minor mass changes.

When the temperature reached 250–500 °C, the TG curve commenced to display a rather distinct downward tendency. This indicated that the epoxy resin initiated rapid thermal decomposition, thereby leading to significant mass loss. On the DTG curve within this specific temperature interval, a prominently sharp peak emerged. Significantly, the temperature corresponding to the peak apex represented the point at which the decomposition rate of the epoxy resin reached its maximum value. Moreover, as presented in Table 2, the initial decomposition temperatures and the maximum decomposition rate temperatures of the epoxy resin both before and after the aging process were tabulated for temperature resistance analysis. The initial decomposition temperatures and the maximum decomposition rate temperatures underwent a process of first increasing, then decreasing, and then increasing again.

In the early stage of UV aging, the energy of UV light can promote the incomplete reactive functional groups in epoxy resin to undergo post-curing reactions. The formation and development of the cross-linked structure restricted the movement of molecular chains, making it more difficult for the molecular chains to displace and break under the action of heat, thus increasing the decomposition temperature. With the extension of UV aging time, the energy of UV light could continuously damage the molecular chain structure of epoxy resin. When the cross-linked structure was damaged to a certain extent, the length and molecular weight of the molecular chains decreased, the intermolecular forces weakened, and the mobility of the chain segments increased, resulting in a decrease in the decomposition temperature. When the UV aging time continues to increase, some new chemical reactions may occur in the epoxy resin system, such as the recombination of free radicals and the recurrence of cross-linking reactions, thus forming a new cross-linked structure. In addition, long-term UV aging may change the surface structure of epoxy resin, such as the formation of a dense oxide layer or carbonized layer. This surface structure can play a certain protective role, preventing the transfer of heat and the further decomposition of molecular chains, thereby increasing the decomposition temperature. Overall, the magnitude of either the decrease or increase in the resin’s degradation temperature was not substantial. The unaged epoxy resin exhibited an initial decomposition temperature of 341.79 °C, while the aged resin for 1080 h showed a slightly higher value of 343.89 °C, indicating a mere increase of 2.1 °C. This is because the thickness of the degradation layer formed by UV radiation was very thin, perhaps only a few hundred microns [28,29], which has little impact on the material properties under the degradation layer.

#### 3.1.6. SEM Analysis

**Figure 10:**
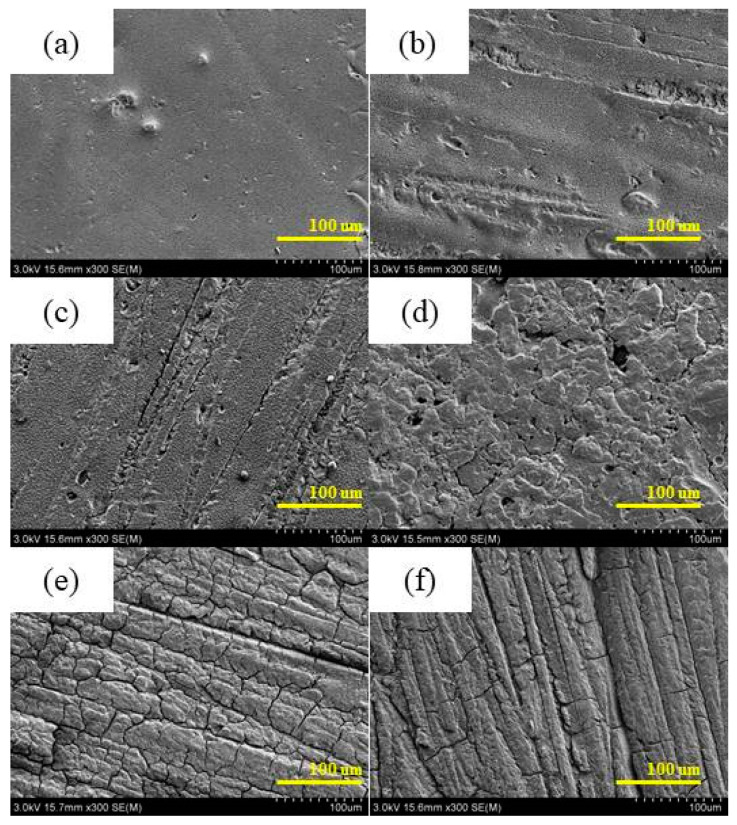
SEM of the surface of epoxy before and after UV aging. (**a**) 0 h; (**b**) 216 h; (**c**) 360 h; (**d**) 792 h; (**e**) 936 h; (**f**) 1080 h.

Figure 10 shows the effect of UV aging on the microstructure of surface epoxy resin. No significant alterations in the surface morphology were observed within the first 216 h of aging, while a few cracks emerged after 360 h of exposure to UV radiation. With prolonged aging, the crack density on the resin’s surface increased and exhibited a network-like distribution.

### 3.2. Ageing Properties of CFRP

Due to the combination of the characteristics of multiple materials, the aging process of the composite is more complex and unique. This study systematically compared the changes in multiple mechanical properties of CFRP (i.e., bending strength and impact strength) with UV aging time and explained the UV irradiation aging mechanism of CFRPs by SEM and FTIR.

#### 3.2.1. Mechanical Properties

**Figure 11:**
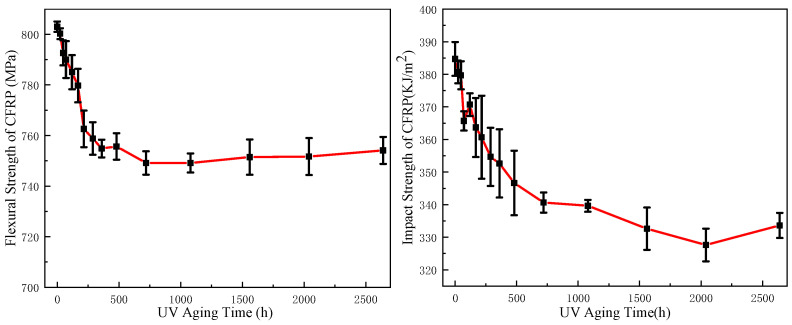
Mechanical property change in CFRP before and after UV aging.

Figure 11 shows the test results of CFRP mechanical properties under different UV aging times. As observed from the figure, both the bending strength and impact strength of the CFRP exhibited a consistent trend with increasing UV aging time. Notably, the property of CFRP deteriorated rapidly in the early stage of aging, and the rate of property decline gradually slowed down in the later stage and gradually stabilized at about 480~720 h. The bending strength and impact strength decreased by 6.0% and 12.8%, respectively, from the initial values of 803 MPa and 384 kJ/m^2^ to 755 MPa and 335 kJ/m^2^.

Under the influence of ultraviolet light, epoxy resin undergoes two main changes, namely the photo-oxygen aging reaction and the post-curing reaction. During the initial stage of UV aging, the effect intensity of the photo-oxygen aging reaction may be higher than that of the post-curing reaction. The degradation of the resin due to aging led to a decline in interface adhesive strength, resulting in reduced mechanical properties. In the later stages, surface-level photo-oxygen aging gradually decelerated as ultraviolet light penetration into the material became less feasible, thereby weakening the photo-oxygen aging reaction. Simultaneously, the post-curing process enhanced heat resistance temperature and increased cross-linking of resin. Consequently, under this combined effect, there was no significant alteration observed in the material’s mechanical strength.

**Figure 12:**
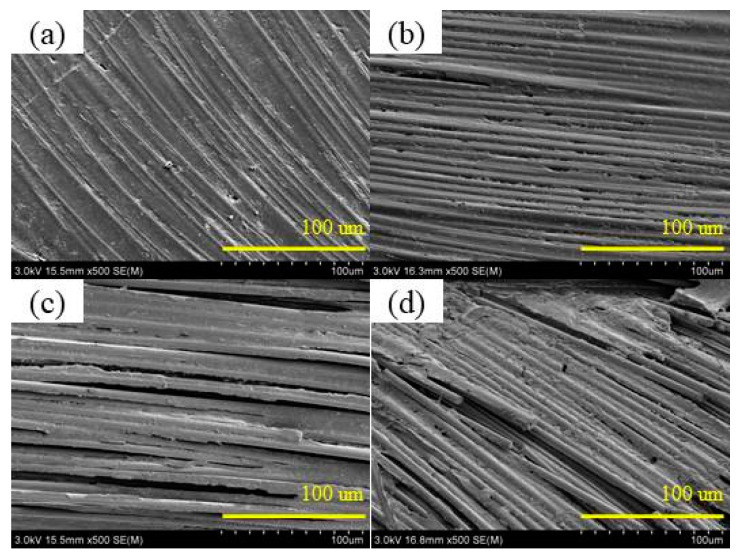
SEM (500×) of the surface of CFRP before and after UV aging. (**a**) 0 h; (**b**) 288 h; (**c**) 1560 h; (**d**) 2640 h.

**Figure 13:**
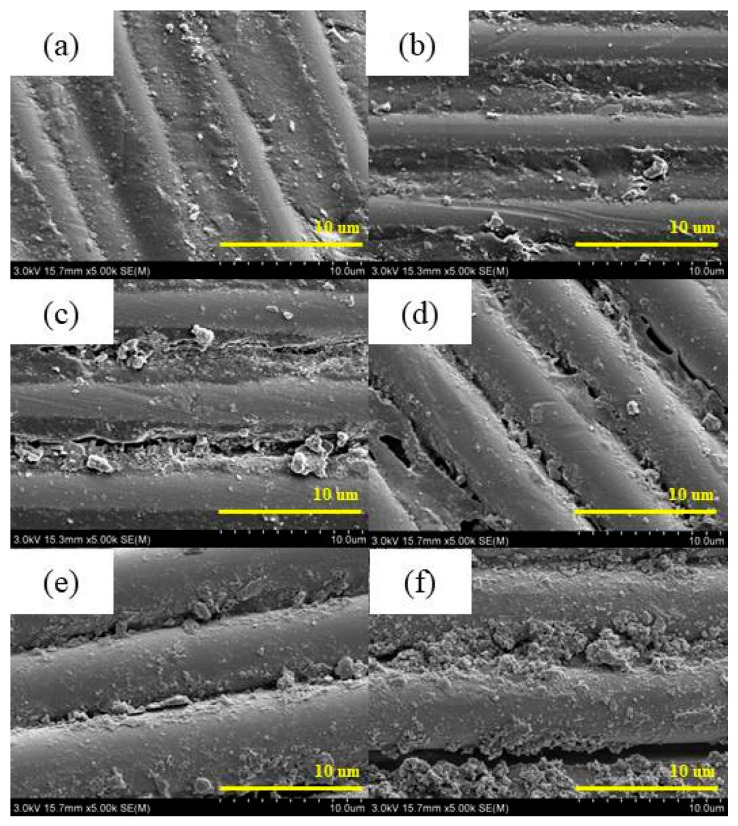
SEM (5000×) of the surface of CFRP before and after UV aging. (**a**) 0 h; (**b**) 240 h; (**c**) 960 h; (**d**) 2160 h; (**e**) 3120 h; (**f**) 5280 h.

The SEM photos shown in Figure 12 and Figure 13 depict the microstructures of the surface layer of CFRP under different UV aging times, magnified at 500× and 5000×, respectively. CFRP exhibited a well-integrated bond between resin and the fibers, displaying a relatively flat and uniform matrix structure before aging in Figure 12a and Figure 13a. After an initial UV aging period of 240 h, no significant changes were observed in the interface bonding between the matrix and fiber components. After 288 h of aging, a slight fragmentation phenomenon was observed in the matrix, accompanied by the emergence of a few surface cracks on the CFRP specimen. As the aging process continued, the matrix degradation became more serious, leading to the resin powdering, and the gaps between the fibers and the matrix became larger.

#### 3.2.2. FTIR Analysis

**Figure 14:**
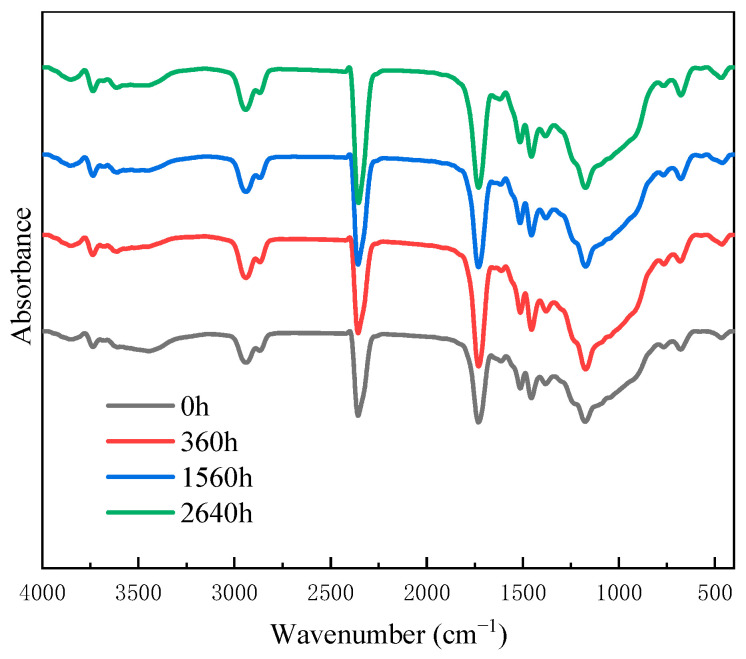
FTIR spectra of CFRP before and after UV aging.

Figure 14 shows the FTIR spectra of CFRP with different UV aging times. The position and relative strengths of the characteristic peaks of CFRP remained largely unchanged before and after UV aging. It was different from the FTIR analysis of epoxy resin described in the previous context. UV radiation only induced degradation of the outermost resin during the testing period. The resin volume fraction of CFRP was approximately 40 vol%, which resulted in weaker infrared spectral signals compared to pure resin. Carbon fiber is a microcrystalline graphite material obtained through pre-oxidation, carbonization, and graphitization processes that exhibits excellent radiation resistance. The outer carbon fibers provided certain shielding effects against ultraviolet radiation and prevented resin exposure to UV irradiation.

Combined with the morphology and FTIR analysis, the main reasons for the deterioration of mechanical properties of CFRP were analyzed as follows: (1) Under UV radiation, the resin underwent chain fragmentation, resulting in the formation of reactive free radicals and subsequently initiating a photo-oxidative aging process with oxygen. Consequently, the degradation of the resin matrix occurred alongside a reduction in cross-linking density. (2) Under UV irradiation, the resin underwent post-curing and cross-linking reactions, thereby enhancing its thermal resistance. However, experimental findings suggested that the effect of photo-oxygen degradation was greater than that of post-curing. (3) The degradation of the resin, combined with periodic UV irradiation and condensation, led to the expansion and contraction of the resin. This resulted in the generation of small defects such as fragments, holes, or cracks in certain areas of the resin, weakening the bond between the fibers and resin and causing interface debonding. Simultaneously, water sprayed during UV aging can penetrate samples through these defective regions. Due to differential expansion coefficients between fibers and resin, absorbed water by the resin-induced shear stress at interfaces further aggravates the debonding phenomena and reduces mechanical properties. (4) UV radiation had limited penetration depth and mainly affected the surface of materials. Glass fiber and carbon fiber have stable properties, which were largely unaffected by UV radiation and provided a certain barrier effect against the UV irradiation energy and defect expansion [12]. This was also why the performance of CFRP significantly deteriorated in the early stage of UV aging but changed little in later stages.

## 4. Conclusions

In this study, the effects of UV aging on the properties of epoxy resin and composites were investigated through artificially accelerated aging experiments. The conclusions were as follows:

(1) UV aging mainly impacted the properties of the surface layer resin in epoxy resin. Under UV radiation, the resin underwent photooxidation, degradation, and cross-linking chemical reactions.
(2) These reactions led to changes in the resin’s weight, density, hardness, and color. The weight change rate and density first increased and then decreased with the increase in aging time. The color deepened, and cracks appeared in the network-like distribution. The heat resistance of the resin showed a slight improvement. The hardness of the resin initially increased, then reached a constant value lower than the non-aging material’s initial value, with a decrease of 1.4%.
(3) Ultraviolet irradiation could also cause the deterioration of CFRP properties. The bending and impact properties of CFRP showed a change law similar to that of the hardness of resin, and the values initially increased, then reached a constant value lower than the non-aging material’s initial value, with decreases of 6.0% and 12.8%, respectively.

This study is highly significant for improving the durability and extending the service life of PMC products in outdoor applications. However, the current study also has certain drawbacks. Firstly, the artificially accelerated aging experiments might not fully mimic the complex and diverse real-world outdoor conditions, which contain a multitude of factors such as fluctuating temperature, humidity, and pollutants, which could interact with UV radiation and potentially have different impacts on the resin and composites compared to the controlled laboratory setting. Secondly, this study mainly focused on the macroscopic properties changes of the materials, while the microscopic mechanisms underlying these changes, such as the detailed molecular structural alterations during UV aging, were not explored in-depth.

Future research could focus on several aspects. More comprehensive outdoor exposure tests could be carried out, in combination with advanced monitoring techniques to continuously track the performance changes of materials in the actual environment. In terms of microscopic mechanisms, advanced spectroscopic and microscopic analysis methods could be employed to investigate the molecular and nanostructural evolution during UV aging, providing a deeper understanding of the degradation and modification processes.

## Figures and Tables

**Table 1:** UV aging conditions.

Exposure	Fluorescent Lamp	Irradiation (W/m^2^ × nm)	Wavelength nm	Temperature °C
Irradiation for 8 h	UV-340	1.55 ± 0.02	340	60 ± 3
Spray for 0.25 h	/	0	/	Uncontrolled
Condensation for 3.75 h	/	0	/	50 ± 3

**Table 2:** Change in temperature resistance of epoxy resin under UV aging.

Aging Time/h	The Initial Degradation Temperature/°C	The Maximum Degradation Rate Temperature/°C
0	341.79	379.67
216	343.26	381.34
792	341.56	378.43
1080	343.89	380.60

## Data Availability

The original contributions presented in this study are included in the article. Further inquiries can be directed to the corresponding author.
